# Risk Factors, Not Low-Osmolar Contrast, Predict Acute Kidney Injury Following Contrast-Enhanced Computed Tomography: A Comparative Retrospective Cohort Study

**DOI:** 10.7759/cureus.108615

**Published:** 2026-05-10

**Authors:** Kyaw K Hoe, Julear Hobson

**Affiliations:** 1 Medicine, University of the West Indies, Kingston, JAM; 2 Medicine, University Hospital of the West Indies, Kingston, JAM

**Keywords:** acute kidney injury, ca-aki, contrast agents, contrast-enhanced ct, iopromide, low-osmolar contrast media

## Abstract

Background: The safety of a low-osmolar contrast agent, iopromide (Ultravist, Bayer, Leverkusen, Germany), currently being used is unknown. This study aimed to evaluate whether iopromide use was associated with acute kidney injury (AKI) in patients undergoing contrast-enhanced computed tomography (CT) imaging.

Methods: This retrospective cohort study included 1915 participants who had CT scans during 2023. AKI was defined as an absolute rise in serum creatinine (sCr) ≥26.5 µmol/L within 48 hours or ≥1.5 times increase from baseline within seven days. The differences in the means of pre-CT and post-CT sCr with and without contrast exposure were determined using the independent samples t-test and variance ratio (F-test). Paired t-test was used to find the mean difference between pre- and post-CT creatinine levels. Pearson's chi-squared (X^2^) test, along with pairwise correlation analysis, was applied to find the relationship between contrast exposure and AKI as well as AKI and renal outcomes. To find out the independent predictors of contrast-associated acute kidney injury (CA-AKI), a multivariable binary logistic regression model was conducted.

Results: Of 1915 participants who underwent CT imaging, 869 (45.4%) were exposed to contrast, while 1046 (54.6%) were not exposed to contrast. No statistically significant difference was found between exposed and non-exposed groups for the development of AKI (10.6% vs. 8.8%; p=0.188). Both groups were found to have lower mean post-procedure sCr compared to pre-procedure sCr (-2.2 and -12.7 µmol/L, respectively). The AKI occurrence was not directly associated with whether patients were exposed to iopromide or not (r=0.03; p=0.178). Noteworthy risk factors identified were active malignancy (aOR: 2.43; 95% CI: 1.54-3.84; p<0.001), pre-existing renal dysfunction (aOR: 2.31; 95% CI: 1.27-4.18; p=0.006), and cardiac disease (aOR: 2.09; 95% CI: 1.22-3.58; p=0.007).

Conclusion: Use of low-osmolar contrast agents for CT imaging was not independently associated with AKI. Our results suggest that contrast-enhanced CT may be considered when clinically indicated without unnecessary delay while acknowledging that individual patient risk assessment remains essential. Greater emphasis should instead be placed on identifying and optimizing certain factors, like active malignancy, pre-existing renal dysfunction, and cardiac diseases, to prevent CA-AKI.

## Introduction

Evidence suggests that the newer contrast agents significantly mitigate the risk of acute kidney injury (AKI) compared to their predecessors, challenging the long-standing doctrine that deemed all contrast agents as equally nephrotoxic. The terminology surrounding this condition has evolved in tandem with this enhanced understanding. Initially termed contrast-induced nephropathy (CIN), it has since been redefined as contrast-induced acute kidney injury (CI-AKI) and more recently as contrast-associated acute kidney injury (CA-AKI) [1]. CI-AKI is specifically characterized as AKI that is attributable to the administration of contrast agents, whereas CA-AKI encompasses AKI that coincides temporally with such administration [2]. This acknowledges the multifactorial aetiologies that may contribute to renal decline in the context of imaging procedures. Despite advancements in study design and data analysis in more recent studies, a significant underrepresentation of Black patients remains a notable challenge. This gap is particularly concerning given the documented higher incidences of AKI among Black patients which was thought to be genetically vulnerable [3].

The underlying pathophysiology of CA-AKI is thought to involve a combination of direct tubular toxicity and ischemic injury [4]. Free iodine and high contrast osmolality can disrupt endothelial function, leading to oxidative stress, vasoconstriction, and medullary hypoperfusion. In addition, increased tubular fluid viscosity may reduce urine flow and prolong exposure to potentially nephrotoxic agents [5]. Regarding iopromide, a low-osmolar contrast agent, an animal study revealed increased glomerular inducible nitric oxide synthase (iNOS) expression for a short term without any changes in renal function after 24, 48, and 72 hours post-administration [6]. In terms of prevalence and incidence, there have been differences in the rates of CA-AKI according to the osmolarity of the contrast used and the cutoff point of absolute serum creatinine (sCr) rise.Some studies used a slightly higher absolute threshold of 0.5 mg/dL (≥44.2 µmol/L) as an indicator of AKI [7].

We hypothesized that there was no association between iopromide exposure and CA-AKI (H_0_). The aim of the study was to evaluate whether the use of a low-osmolar contrast agent (iopromide) was associated with AKI. The primary objective was to determine the association between the low-osmolar contrast agent and AKI. The secondary objectives were to explore the risk factors and outcomes of renal recovery, length of hospitalization, requirement of dialysis, and mortality in CA-AKI. The findings might help nephrology services to give accurate input on seeking consultations.

## Materials and methods

Methods

We conducted a retrospective cohort study with participants who underwent computed tomography (CT) at the University Hospital of the West Indies (UHWI) in Kingston, Jamaica. The Picture Archiving and Communication System (PACS) was used to retrieve CT scans performed by the Radiology Department of the UHWI during the period January 1, 2023, to December 31, 2023. Minors under 18 years old and patients with end-stage renal disease were excluded. We also excluded patients who underwent major surgery within five days of CT imaging. This exclusion criterion was prespecified to eliminate the confounding effect of postoperative elevation of sCr due to inevitable tissue injuries. The study analyzed patient records from a 12-month period spanning January 31, 2023, to December 31, 2023. Following institutional ethics approval from the Research Ethics Committee of the University of the West Indies (approval number: CREC-MN.0121,2023/2024) on May 29, 2024, the data access and retrieval window occurred between June 1, 2024, and September 30, 2024. Data analysis was subsequently performed from October to December 2024. Records were then cross-referenced against the electronic medical records system to identify the scans that were performed on adult patients admitted to the UHWI.

Materials

Patients who underwent contrast-enhanced CT scans received a non-ionic low-osmolar contrast agent (iopromide (Ultravist, Bayer, Leverkusen, Germany) 300 or 370 I/ml) with a standard dose of 100 ml intravenously; however, the exact dose administered to each patient was not available. The standardized hydration protocol was used for all patients. sCr measurements were performed at the Department of Chemical Pathology of the UHWI using the Roche c111 analyzer (Basel, Switzerland), an automated chemistry analyzer. Creatinine quantity was determined using the Jaffé Gen.2 method, standardized to the isotope dilution mass spectrometry (IDMS) reference method. Internal quality control procedures were performed according to the manufacturer's recommendations to ensure analytical reliability and consistency throughout the study period. The race-free 2021 Chronic Kidney Disease Epidemiology Collaboration (CKD-EPI) equation was used to get the estimated glomerular filtration rate (eGFR) [8], and the calculation was performed using Stata (StataCorp LLC, College Station, Texas, United States). Presence of comorbidities was defined as physician-documented hypertension, diabetes mellitus, renal dysfunction, liver cirrhosis, active malignancy, and cardiac diseases (mainly cardiac failure and acute coronary syndrome). AKI was defined based on the Kidney Disease Improving Global Outcomes (KDIGO) guidelines with an increase in sCr ≥26.5 µmol/l within 48 hours or an increase in sCr to ≥1.5 times from baseline occurring within the preceding seven days [9]. The urine output criterion was not applied as this data was not readily available in health records. Complete renal recovery was defined as a return of sCr to ≤ baseline + 26 µmol/L (0.3 mg/dL) during the admission or at discharge. Baseline creatinine was defined as the most recent stable pre-event value. The Strengthening the Reporting of Observational Studies in Epidemiology (STROBE) guidelines for cohort studies were followed when reporting results [10]. 

The estimation of sample size was calculated based on a review of existing literature. The Fleiss formula for comparing two proportions was applied. With an anticipated prevalence of AKI of 6% in the contrast-exposed group [11] and 3% in the non-exposed group, we determined that 1,496 patients were needed minimally to detect a significant association with a power of 90% and an α of 0.05.

Statistical analysis

All data analyses were conducted using Stata Version 18. Collected variables included age, sex, blood pressure, comorbidity profiles, pre- and post-CT sCr, hemoglobin, dialysis initiation, in-hospital mortality, and length of hospital stay.

Continuous variables were summarized using mean and standard deviation if normally distributed and median, when normality deviates, while categorical variables were summarized using frequency and percentages. Descriptive analysis was conducted using the independent samples t-test and Mann-Whitney U test according to the normality of distribution. Changes in sCr were assessed via the paired Student's t-test.

Bivariate association between contrast-exposed and non-exposed groups and comparison analysis of categorical variables, gender, comorbidity, and development of AKI were analyzed using Pearson's chi-squared test. Fisher's exact test was applied for the association between two categorical variables, chronic kidney disease (CKD) stage 3/CKD stage 4 and CA-AKI, as the sample size of the subgroup analysis was very small. One-way analysis of variance (ANOVA) was also used to assess relationships between categorical and continuous variables, as needed. Pairwise correlation analysis was performed using Pearson's or Spearman's correlation coefficient between contrast exposure and CA-AKI. Differences in hospital length of stay were evaluated using variance ratio analysis. The baseline differences were addressed using multivariable adjustments. The significant baseline differences, such as pre-existing renal impairment/CKD, cardiac diseases, and active malignancy, along with age category and gender were included as covariates in the multivariate logistic regression model to control the sicker profile of the non-exposed group, and adjusted odds ratios (aOR) were calculated to isolate the effect of contrast exposure. Pearson's proportional analysis was applied to get the relative risk of renal recovery, requirement of dialysis, and mortality between CA-AKI and non-CA-AKI. The strength of association between contrast exposure and outcomes (AKI, dialysis, mortality) was expressed as odds ratios (ORs) with 95% confidence intervals (CI). Statistical significance was defined as p<0.05.

## Results

**Figure 1:**
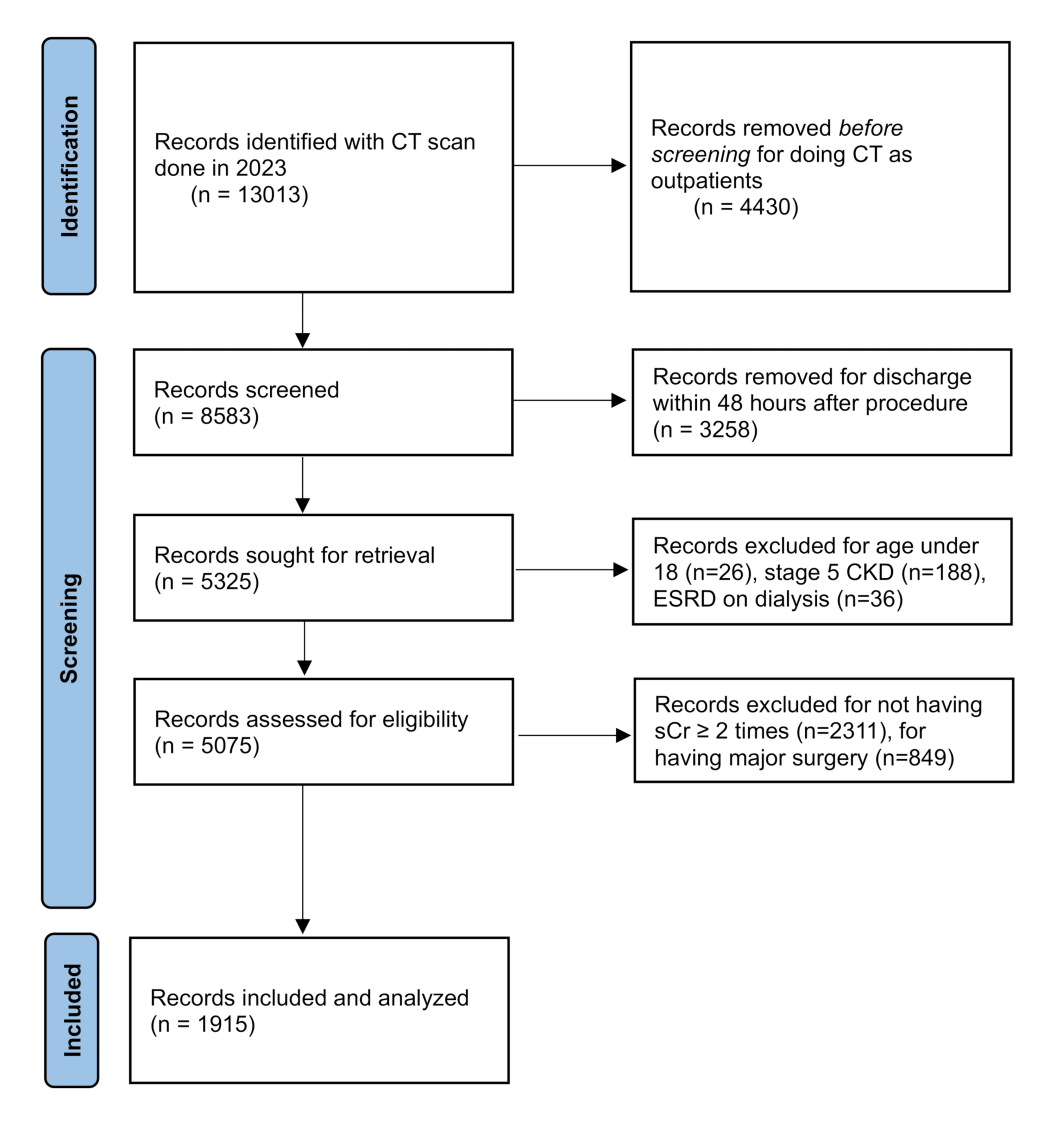
Cohort selection flow diagram of the study selection process The flowchart delineates the stages of identification, screening, eligibility, and inclusion for the cohort. The GIMP 3.0.8 (GIMP Development Team, University of California, Berkeley, Berkeley, California, United States) was used for the creation of the image. CT: computed tomography; CKD: chronic kidney disease; ESRD: end-stage renal disease; sCr: serum creatinine

Of 13,013 CT scans performed, a total of 7,688 CT scans were excluded as these were performed on either outpatients, privately admitted patients, or patients seen in the emergency room and discharged within 24-48 hours without repeat sCr measurements. Further exclusions were done for patients who were under age 18 (N=26), underwent surgery during hospital admission (N=2,311), or had CKD stage 5 or were on renal replacement therapy (N=224). The electronic lab information system (LIS) was then used to further exclude 849 CT scans that did not have an accompanying sCr measurement either prior to CT or up to one week post-CT scan. Patients may have been included in this study more than once if each CT scan met the inclusion criteria (Figure 1).

The selected 1,915 patients who underwent CT imaging had a mean age of 66.2±16.4 years, with a balanced gender distribution (p=0.784). AKI was observed in 92 (8.8%) in the contrast-exposed group and 92 (10.6%) in the non-contrast-exposed group (p=0.188). Types of CT scans were collectively described in Table 1.

**Table 1:** Distribution of types of CT scans performed and contrast exposures (n=1,915) The chi-squared (X^2^) test of independence was used to get the frequencies (%) of categorical variables between two independent groups. ^a ^Values are presented as frequency (n) and percentage (%). ^b^ include CT angiography and multiphasic scans. CT: computed tomography; HRCT: high-resolution computed tomography, KUB: kidney, ureter, and bladder

Type of CT imaging^b^	Total^a^, n (%)	Non-contrast exposure^a^, n (%)	Contrast exposure^a^, n (%)
	1,915 (100)		
CT of the brain	966 (50.4)	933 (48.7)	33 (1.7)
CTPA	420 (21.9)	0 (0)	420 (21.9)
CT of the abdomen	262 (13.7)	35 (1.8)	227 (11.9)
CT of the chest/HRCT	49 (2.6)	6 (0.3)	43 (2.2)
CT aortogram/angiogram	35 (1.8)	0 (0)	35 (1.8)
CT of the spine	25 (1.3)	22 (1.1)	3 (0.2)
CT KUB/urogram	34 (1.8)	21 (1.1)	13 (0.7)
CT of the face/limb/joint	55 (2.9)	7 (0.4)	48 (2.5)
CT > one area	69 (3.6)	22 (1.1)	47 (2.5)

Baseline characteristics of the contrast-exposed and non-exposed participants

The mean age of the contrast-exposed participants was 61.5±19.7 years which was significantly younger than the mean age of the non-exposed participants (68.0±18.6 years) (p=0.001). Patients were subcategorized by age: young age, 16-40 years (13.2%; n=253); middle age, 41-65 years (31.6%; n=605); and elderly, >65 years (55.2%; n=1,057). A trend was observed where pre-CT sCr levels increased with age, rising from a mean of 92 µmol/L in the youngest group to 118.9 µmol/L in the middle-aged group and 126.4 µmol/L in patients over 65. Post-CT mean creatinine levels followed a similar pattern: 91.1 µmol/L in young patients, 113.1 µmol/L in middle-aged patients, and 115.4 µmol/L in elderly patients.

As regards gender, 1,049 participants (54.8%) were females, and 866 (45.2%) were males. Female distribution was 54.5% in the non-exposed group and 55.1% in the exposed group, while male distribution was 45.5% in the non-exposed group and 44.9% in the exposed group. There was no difference in gender distribution between the exposed and non-exposed groups (p=0.784). Demographic outcomes are summarized in Table 2.

**Table 2:** Baseline demographic and clinical characteristics of eligible patients undergoing CT scan with and without contrast at the UHWI Radiology Department during the year 2023 Data are presented as mean±standard deviation (SD) and p-value which were calculated using the independent samples t-test, chi-squared test, and Mann-Whitney U test based on normality. Pre-existing RD is defined as eGFR <60 ml/min/1.73 m^2^. CT: computed tomography; BP: blood pressure; sCr: serum creatinine; eGFR: estimated glomerular filtration rate; RD: renal dysfunction; AKI: acute kidney injury; UHWI: University Hospital of the West Indies

Demography	Total, n (%)	Non-contrast CT group, n (%)	Contrast CT group, n (%)	95% CI	P-value
	1,915 (100)	1,046 (100)	869 (100)		
Age (mean±SD)	65.05±19.38	68.00±18.64	61.50±19.67	0.015-0.044	<0.001
Gender					
Female	1,049 (54.8)	570 (54.5)	479 (55.1)	0.814-1.168	0.784
Male	866 (45.2)	476 (45.5)	390 (44.9)		
Systolic BP (mean±SD mmHg)	134±25	138±26	128±24	136.76-139.9	<0.001
Diastolic BP (mean±SD mmHg)	78±20	80±24	76±14	78.5-81.4	<0.001
Hemoglobin (mean±SD g/dL)	11.5±4.7	12.1±5.7	10.8±2.9	11.8-12.5	<0.001
Pre-study sCr (mean±SD µmol/L)	119±102	133±118	103±75	125.7-140	<0.001
Pre-study eGFR (mean±SD ml/min)	65±32	60±32	72±31	58.03-73.9	<0.001
Post-study sCr	112±101	121±115	101±79	113-127	<0.001
Post-study eGFR (mean±SD ml/min)	73±32	69±32	78±32	67-80	<0.001
Received IV fluid	1,101 (57.5)	585 (55.9)	516 (59.4)	0.72-1.04	0.128
Comorbidities					
Hypertension	990 (51.7)	616 (58.9)	374 (43)	1.58-2.27	<0.001
Diabetes	567 (29.6)	356 (34)	211 (24.3)	1.32-1.97	<0.001
Pre-existing RD	146 (7.6)	96 (9.2)	50 (5.8)	1.16-2.36	0.005
Chronic liver disease	20 (1)	9 (0.9)	11 (1.3)	0.28-1.64	0.385
Cardiac diseases	241 (12.3)	129 (12.3)	112 (12.9)	0.73-1.25	0.715
Malignancy	364 (19)	127 (12.1)	237 (27.2)	0.29-0.47	<0.001
Length of hospital stay (days)	14.5±17.6	12.8±16.3	16.7±18.9	0.004-0.02	<0.001
AKI	184 (9.6)	92 (8.8)	92 (10.6)	0.60-1.10	0.183

Post-CT imaging sCr changes

**Figure 2:**
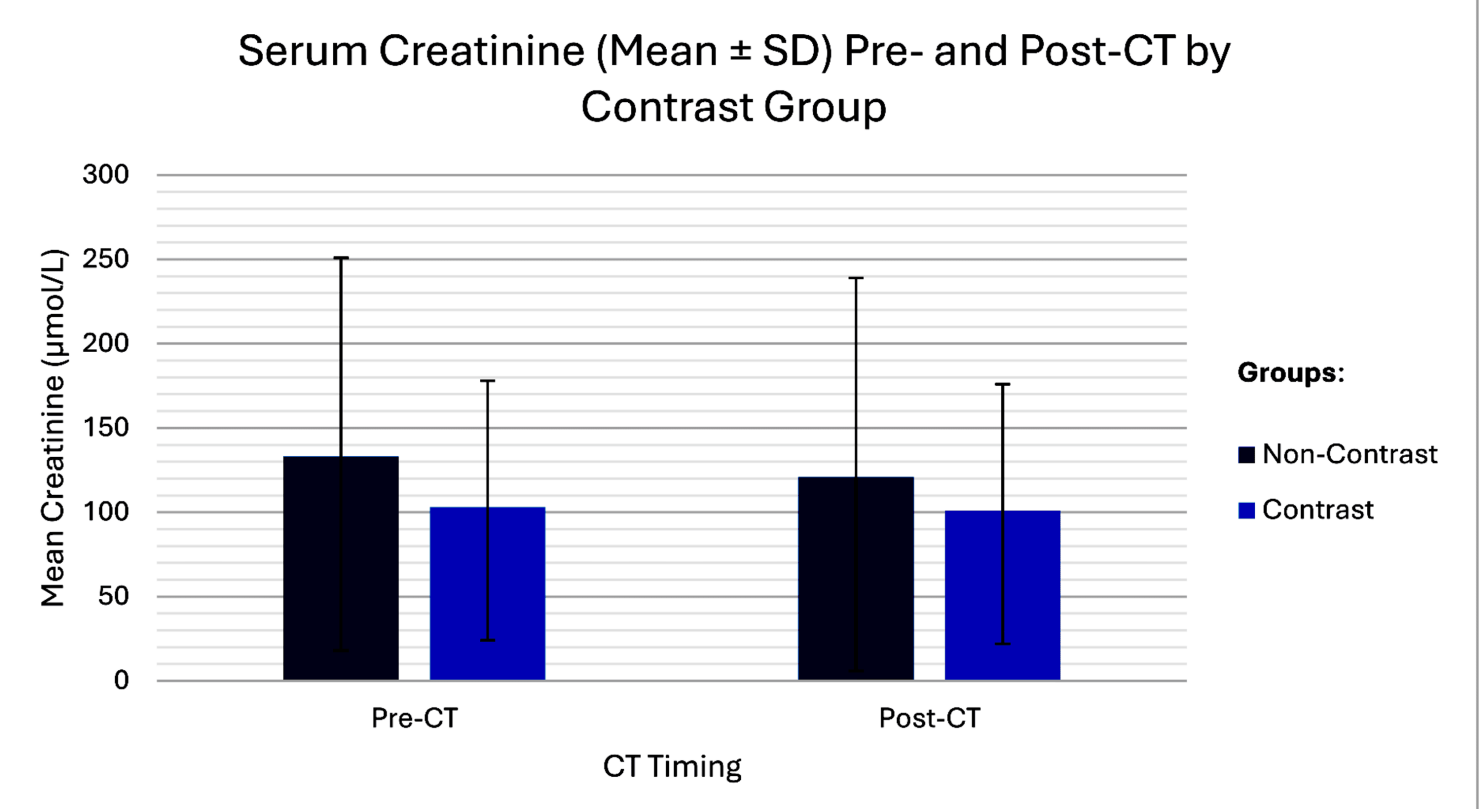
Comparison of sCr levels before and after CT scan Box-and-whisker plots represent the distribution of sCr concentrations (µmol/L) at baseline (pre-CT scan) and at peak levels within five days post-CT scan. The vertical lines represent the spread of the data outside the middle 50%. sCr changes reveal a higher drop in the non-exposed group post-CT, compared to the contrast-exposed group. The GIMP 3.0.8 (GIMP Development Team, University of California, Berkeley, Berkeley, California, United States) was used to generate the image. CT: computed tomography; sCr: serum creatinine

**Figure 3:**
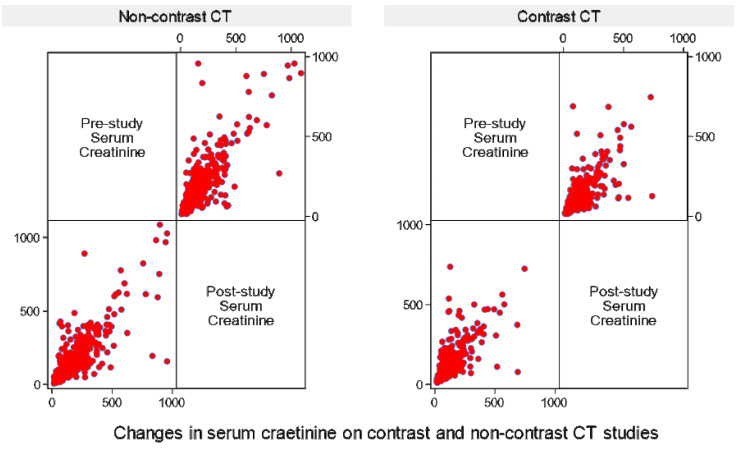
Comparison of sCr levels before and after contrast administration The scatter plot revealed no significant difference in sCr before and after CT whether patients were non-exposed to the contrast or exposed to the contrast (pre-CT sCr: 133±118 vs. 103±75 µmol/L (p<0.001); post-CT sCr: 121±115 vs. 101±79 µmol/L (p<0.001)). The top-right panel displays the pre-CT levels, while the bottom-left panel displays post-CT level. For each respective plot, both the x-axis and y-axis represent the sCr values for that specific time point, allowing for the visual assessment of data density and distribution within each phase of the study. The GIMP 3.0.8 (GIMP Development Team, University of California, Berkeley, Berkeley, California, United States) was used to generate this image. CT: computed tomography; sCr: serum creatinine

Baseline sCr levels were 103±75 µmol/L (eGFR=72±31 ml/min/1.73 m^2^) in the contrast-exposed cohort and 133±118 µmol/L (eGFR=60±32 ml/min/1.73 m^2^) in the non-exposed group. Both groups were found to have lower mean post-procedure sCr compared to pre-procedure sCr. Participants who were exposed to contrast had an average sCr drop of -2.2 µmol/L, and those who were not exposed had an average drop of -12.7 µmol/L. The difference in mean changes was 10.5 µmol/L, and it was statistically significant (p<0.001), indicating that participants who were not exposed to contrast had a significantly larger drop in sCr. A variance ratio (F-test) showed a significantly higher sCr variability in the non-contrast group (SD=116.8 µmol/L) compared to the contrast group (SD=78.3 µmol/L) (F=2.22; p<0.0001), and post-CT values maintained a similar pattern (SD=113.8 vs. SD=81.6 µmol/L; F=1.94; p<0.0001) (Figures 2-3).

Incidence of CA-AKI and the association between contrast exposure and AKI in the study population

**Figure 4:**
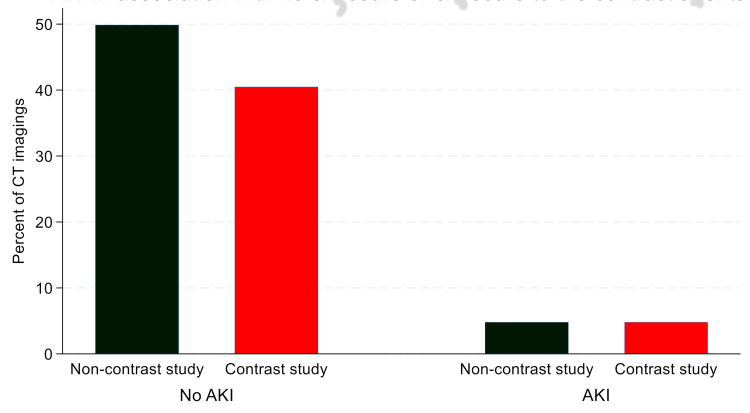
Incidence of AKI in patients with and without contrast-enhanced CT scans Comparative bar chart showing the percentage of participants who developed AKI according to the KDIGO criteria. The contrast CT group (n=869) is compared against a control group of non-contrast CT patients (n=1,046). No significant differences was observed between groups (r=0.03; p=0.178), suggesting multifactorial aetiologies for renal decline in this cohort. The GIMP 3.0.8 (GIMP Development Team, University of California, Berkeley, Berkeley, California, United States) was used to create this image. CT: computed tomography; AKI: acute kidney injury; KDIGO: Kidney Disease Improving Global Outcomes

Among all patients, AKI occurred in 184 patients (9.6%). CA-AKI occurred in 92 patients (4.8%) (Figure 3). The incidence of CA-AKI was 10.6%. The pairwise correlation analysis revealed that the AKI occurrence was not directly associated with exposure to contrast (r=0.03; p=0.178) (Figure 4).

Comorbidities and AKI

**Figure 5:**
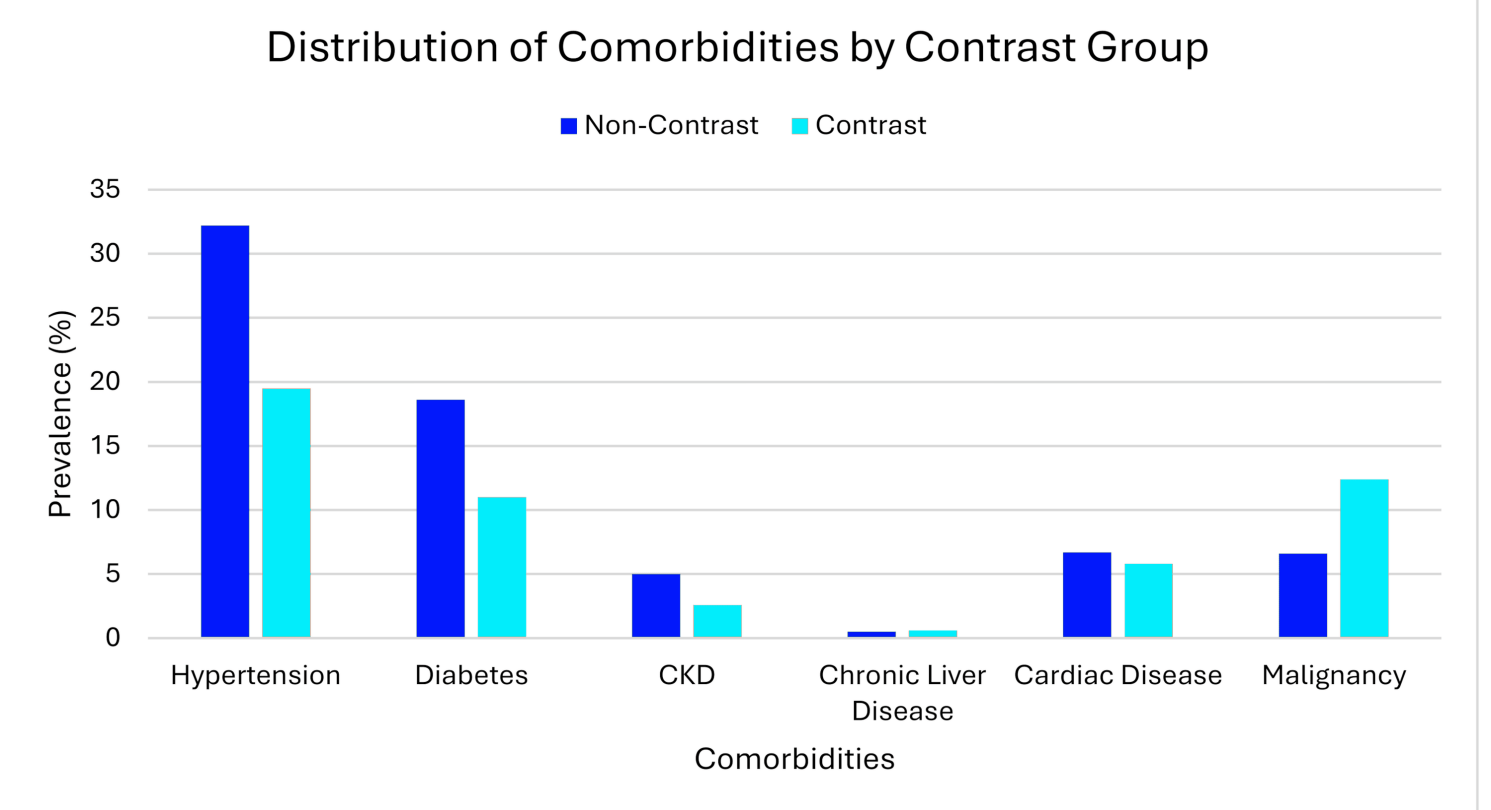
Prevalence of baseline comorbidities among the study population (N=1,915) The bar chart illustrates the percentage of participants affected by chronic conditions at the time of admission. Common comorbidities include hypertension, diabetes, and CKD, and they had less exposure to contrast, while patients with malignancies had more contrast CT scans. The GIMP 3.0.8 (GIMP Development Team, University of California, Berkeley, Berkeley, California, United States) was used for the creation of the image. CT: computed tomography; CKD: chronic kidney disease

The most prevalent comorbidities were hypertension (67.2%), diabetes mellitus (33.6%), pre-existing renal dysfunction (9.1%), cardiac disease (12.8%), and malignancy (14.9%). The lower frequency of contrast exposure was observed in patients with comorbidities compared with patients without comorbidity: hypertension (19.5% vs. 32.2%; p<0.001), diabetes mellitus (11% vs. 18.6%; p<0.001), and CKD (2.6% vs. 5%; p=0.005). Conversely, patients with malignancy had a higher frequency of exposure to the contrast (12.4% vs. 6.6%; p<0.001) (Figure 5).

Comorbidities and CA-AKI

Among contrast-exposed patients with pre-existing renal dysfunction with baseline eGFR 15-59 ml/min/1.73 m^2^, the incidence of CA-AKI was compared across eGFR categories using Fisher's exact test and logistic regression. Of 50 patients with pre-existing renal dysfunction in the contrast-enhanced CT group, 33 had eGFR of 30-59 ml/min/1.73 m^2^ (equivalent to CKD stage 3), and 17 had eGFR of 15-29 ml/min/1.73 m^2^ (stage 4 CKD). In comparison of CKD stage 3 and stage 4 groups, CA-AKI developed in eight (24.2%) of stage 3 patients and eight (47.1%) of stage 4 patients (RR: 1.94; 95% CI: 0.88-4.26; p=0.121). Univariable logistic regression analysis showed a significant association between CA-AKI and pre-existing renal dysfunction (p<0.001), cardiac diseases (p=0.003), and malignancy (p<0.001) (Table 3). 

**Table 3:** Unadjusted (crude) OR for the association between baseline comorbidities and the development of CA-AKI P-values indicate the significance of the association between the specific comorbidity and CA-AKI incidence. ^b^ is defined as a history of acute coronary syndrome and heart failure. Note: Bold values indicate statistical significance (p<0.05). CA-AKI: contrast-associated acute kidney injury

Comorbidities	CA-AKI, n (%)	OR	95% CI	P-value
Hypertension	49 (53.3)	1.07	0.70-1.62	0.758
Diabetes	31 (33.7)	1.22	0.78-1.90	0.379
Pre-existing renal dysfunction	16 (17.4)	2.74	1.55-4.84	<0.001
Liver disease	0 (0)	NA	NA	NA
Cardiac disease^b^	21 (22.8)	2.15	1.30-3.58	0.003
Active malignancy	31 (33.7)	2.27	1.45-3.56	<0.001

The multivariable logistic regression was performed to identify independent risk factors for CA-AKI. The final model included demographic covariates (age and gender) and clinical covariates (pre-existing renal dysfunction, cardiac diseases, and active malignancy) that demonstrated a univariate association of p<0.2. The analysis demonstrated that pre-existing renal dysfunction (aOR: 2.31; 95% CI: 1.27-4.18; p=0.006), cardiac diseases (aOR: 2.09; 95% CI: 1.21-3.58; p=0.007), and active malignancy (aOR: 2.43; 95% CI: 1.54-3.84; p<0.001) remained as independent predictors for CA-AKI and others lost significance, likely due to confounding (Table 4). 

**Table 4:** Multivariable logistic regression model with aOR for the association between comorbidities and CA-AKI ^a^ aOR was adjusted for age, gender, pre-existing renal dysfunction, cardiac diseases, and active malignancy. Pre-existing renal dysfunction, cardiac diseases, and active malignancy remained statistically significant (p<0.05), indicating as independent predictors of CA-AKI. CA-AKI: contrast-associated acute kidney injury

Variable	aOR^a^	P-value	95% CI
Pre-existing renal dysfunction	2.31	0.006	1.27-4.18
Cardiac diseases	2.09	0.007	1.21-3.58
Active malignancy	2.43	<0.001	1.54-3.84
Age categories	0.82	0.548	0.44-1.55
Gender	0.89	0.581	0.58-1.36

Outcomes of AKI in the study

**Figure 6:**
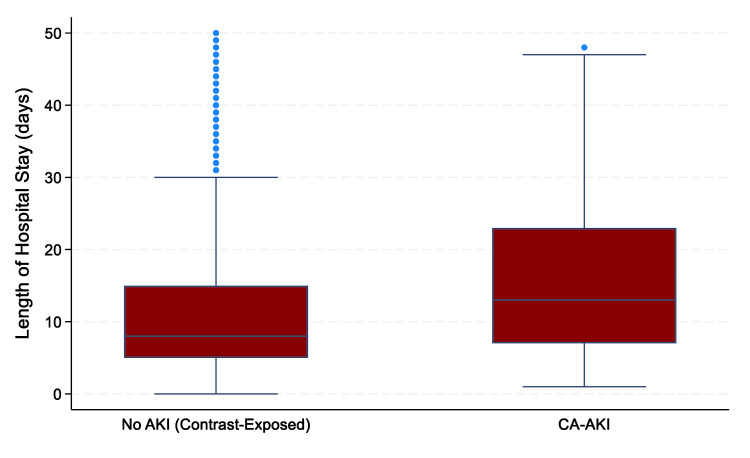
Impact of CA-AKI on hospital length of stay Comparison of the total number of inpatient days between participants with AKI and without AKI among the contrast-exposed group (n=869). Participants in the CA-AKI group exhibited a significant longer median (IQR) of hospital stay (14 days (7-23.5 days) vs. 10 days (5.5-20 days); p=0.042], highlighting the clinical burden of renal complications. The GIMP 3.0.8 (GIMP Development Team, University of California, Berkeley, Berkeley, California, United States) was used for the creation of the image. CA-AKI: contrast-associated acute kidney injury; AKI: acute kidney injury

AKI occurred in 184 (9.6%) of the total participants, of whom 92 (4.8% of the total cohort) fulfilled the criteria for CA-AKI. Complete renal recovery was observed in 41 (39.6%) in the CA-AKI group and 32 (34.7%) in the non-CA-AKI group (RR: 1.28; 95% CI: 0.83-2.72; p=0.175). Dialysis was initiated in six cases (6.5%) in the CA-AKI group and two cases (2.1%) in the non-CA-AKI group. CA-AKI had three times higher risk of the requirement of dialysis, but it was not statistically significant (RR: 3.00; 95% CI: 0.62-14.48; p=0.148). All-cause mortality rate was significantly high in CA-AKI patients compared to non-CA-AKI patients who were exposed to contrast (57.6% vs. 15.5%; p<0.001). However, in-hospital all-cause mortality between the CA-AKI group and AKI in the non-contrast CT group was 57.6% vs. 55.4% (RR: 1.04; 95% CI: 0.61-1.95; p=0.766), suggesting no significant association between contrast exposure and mortality in AKI patients. Among contrast-exposed patients, the median (interquartile range) length of hospital stay was higher in the CA-AKI group compared to those without AKI (14 days (7-23.5 days) vs. 10 days (5.5-20 days); p=0.042; Mann-Whitney U test) (Figure 6).

Effect of prophylactic IV fluid administration

Among patients who developed AKI, pre-contrast IV hydration was recorded in 105 cases (57.1%). Of these, 49 were in the non-CA-AKI group (26.6%) and 56 in the CA-AKI group (30.4%). Despite its routine use, pre-procedural IV fluid administration did not demonstrate a statistically significant protective effect against the development of CA-AKI (OR=0.94; 95% CI: 0.796-1.115; p=0.502), suggesting that hydration alone may not be a protective measure of CA-AKI.

## Discussion

Despite being at risk, use of iopromide, a low-osmolar contrast media, was not independently associated with AKI after adjustment in this study. This finding agreed with the previous studies by McDonald et al. [12] and Hinson et al. [13] and a meta-analysis by Obed et al. [14] which concluded that the risk of AKI following contrast-enhanced CT scans was not significantly increased in contrast-exposed groups, compared to controls. A large retrospective study of Wilhelm-Leen et al. found identical rates of AKI in hospitalized patients who received contrast (5.5%) and who did not (5.6%), after adjustment for confounders [15].

We observed that post-CT reduction in both contrast and non-contrast groups may reflect multiple contributing factors beyond contrast exposure itself, including regression to the mean, supportive clinical management such as hydration, hemodynamic stabilization, or measurement variability. According to hospital protocol, all patients were encouraged to maintain oral hydration following the contrast-enhanced procedure, and patients who were identified as high risk received IV fluid before and after the procedure. These supportive measures may partially explain the improvement in renal indices observed across groups.

In terms of underlying comorbidities, the multivariable logistic regression model identified cardiac diseases, active malignancies, and pre-existing CKD as independent predictors for CA-AKI, while other factors such as advancing age, gender, low systolic blood pressure, hypertension, and diabetes mellitus were not associated with AKI after adjustment for collinearity and confounders. The robust relationship between cardiac comorbidity and renal injury following contrast exposure has been reported by Hinson et al., who observed a 2.24-fold increased risk of CA-AKI in cardiac patients undergoing CT imaging [13].

The increased vulnerability of cancer patients to renal injury following contrast exposure is thought to be multifactorial. This population frequently encounters nephrotoxic medications such as chemotherapy, broad-spectrum antibiotics, nonsteroidal anti-inflammatory drugs (NSAIDs), and diuretics which may compound their renal risk [16]. Active malignancy had a twofold increased risk of CA-AKI compared to patients without malignancy in our study. Notably, this increased risk was observed despite the likelihood that malignancy-related cachexia may lower baseline sCr levels, potentially obscuring underlying renal dysfunction [17]; thus, the observed rise in creatinine was particularly significant in this population. Findings from international studies support and contextualize our results. Hong et al. [18] reported a CA-AKI prevalence of 8% among hospitalized cancer patients undergoing contrast-enhanced CT with near-normal baseline renal function. Similarly, Cicin et al. [19] noted a CA-AKI rate of 20% in a cohort of hospitalized cancer patients in Turkey. Latcha et al. offered additional insight into the dynamics of CA-AKI in oncology populations in their retrospective analysis which reported that cancer patients with eGFR <59mL/min/1.73 m^2^ had an increased rate of AKI, independent of contrast exposure [20].

Similarly, we found significant increased risk of CA-AKI among patients with pre-existing renal dysfunction. Patients with eGFR 15-29 ml/min/1.73 m^2^ had a nearly double risk of developing CA-AKI compared to those with eGFR 30-59 ml/min/1.73 m^2^. The p-value remained above the standard threshold of 0.05 in comparison to these two stages, which indicates that we cannot completely rule out that this observation occurred by chance within this small sample size. Our finding is consistent with previous studies emphasizing the role of renal function in determining susceptibility. For instance, in a multicenter retrospective analysis by Lee et al., the likelihood of AKI was approximately doubled among individuals with advanced renal dysfunction (eGFR <30 mL/min/1.73 m²), independent of diabetes status [21]. Gorelik et al. also found a significantly higher risk of post-contrast AKI among patients with advanced chronic kidney disease [22]. We acknowledge that our subgrouping to CKD stages 3 and 4, to maintain statistical power and stability due to the limited number of participants with pre-existing CKD exposed to contrast-enhanced CT, might result in lesser granular insight into renal risk stratification, compared to analyses on further division of eGFR to 15-29, 30-44, and 45-59 ml/min/1.73 m^2^. We recommend further future studies with a better number of contrast-exposed advanced CKD participants to explore more detailed CKD stratification.

In our analysis, diabetes mellitus did not emerge as a significant risk factor for developing CA-AKI. This aligns with results from a multicenter retrospective study [21] which found that contrast exposure did not significantly increase the risk of AKI among diabetic individuals, except in those with moderate renal impairment (eGFR 30-44 mL/min/1.73 m²). On the other hand, in a West African cohort, Sonhaye et al. found diabetes to be the only independent predictor with a notably higher odds ratio (OR=6.26) although it used a higher diagnostic threshold of absolute sCr rise ≥0.5 mg/dL (44.2 µmol/L) [23].

Underlying hypertension also did not appear to confer an increased risk for developing CA-AKI in our study. Contrary to that, Lun et al. concluded that hypertension was an independent risk factor, but most of the included studies involved intra-arterial contrast administration during emergency percutaneous coronary intervention, a setting markedly different from diagnostic CT scans [24]. Given these contextual differences and the lack of robust data on hypertension in the setting of IV contrast, our findings contribute novel insight by suggesting that hypertension alone may not be a significant driver of CA-AKI risk in hospitalized patients.

Despite the widely held belief, our analysis did not reveal a statistically significant relationship between patient age and the occurrence of CA-AKI. A study conducted in Lebanon found that individuals aged 65 and older were 1.6 times more likely to develop CA-AKI than younger patients [25]. Additional support for this trend comes from Palli et al., who observed a higher rate of renal dysfunction following contrast exposure among older patients in critical care settings [26]. Meta-analyses by Song et al. showed that the incidence of AKI among older adults undergoing contrast-enhanced imaging exceeded 13% [27].

Prevalence and incidence of CA-AKI

The prevalence of CA-AKI was 4.8%, and the incidence was 10.6% in our cohort. A single-center study by Sonhaye et al., with a comparable race and type of contrast to ours, reported a CA-AKI prevalence of 3% [23]. That lower prevalence than our study was likely due to a higher cutoff point of defining AKI (sCr rise >0.5 mg/dL or >44.2 µmol/L). A comparable trend was also observed in a multicenter analysis which documented a 6.8% prevalence of CA-AKI [20]. Their patient cohort received low-osmolar contrast media, and AKI diagnosis was based on KDIGO criteria, closely aligning with our study's methodology. Likewise, Ribeiro et al. reported similar outcomes in a prospective cohort study of Brazilian inpatients [28]. When applying the KDIGO criterion of a ≥0.3 mg/dL rise in sCr, they noted AKI rates of 10.1% in contrast-exposed individuals and 12.4% in those unexposed. Remarkably, the incidence of CA-AKI was extremely low in our study which strongly reinforced the renal safety of low-osmolar contrast use unless certain risk factors coexist.

Renal recovery, hospital stay, requirement of dialysis, and mortality following CA-AKI

In our cohort, renal function recovery rates were similar to those with CA-AKI and those with non-CA-AKI, and both groups had some incomplete renal recoveries. Sonhaye et al. reported complete resolution of CA-AKI in all affected patients in their prospective study of emergency room CT scans [23]. 

Patients who received contrast-enhanced imaging in our study had notably prolonged hospitalizations. Unfortunately, our study did not capture the timing of imaging relative to hospital admission, limiting our ability to determine causality between contrast exposure and prolonged length of stay. Without this temporal data, it remains unclear whether contrast use and CA-AKI directly contributed to extended hospitalization.

Requirement for dialysis had no difference between CA-AKI and non-CA-AKI, and very minimal numbers in both groups needed dialysis. Williams et al. similarly found no significant increase in dialysis risk among CA-AKI patients compared to controls [29].

Although mortality was high among patients with CA-AKI, similar high all-cause mortality was also observed in AKI patients without contrast exposure in our study, suggesting that the excess mortality is more likely attributable to AKI itself and the underlying severity of illness rather than contrast administration alone. These findings indicate that AKI may serve more as a marker of critical clinical status than as a direct consequence of contrast exposure. It is in agreement with the meta-analysis by Obed et al. who found no meaningful disparity in mortality between contrast-related and non-contrast-related AKI across multiple studies [14].

Effectiveness of pre-procedural IV hydration

Our study did not reveal a statistically significant reduction in CA-AKI among patients who received IV fluids prior to contrast-enhanced imaging, suggesting that hydration alone may not fully mitigate AKI risk in the vulnerable population. Hydration was thought to mitigate CA-AKI by diluting contrast concentration within renal tubules and decreasing fluid viscosity. However, with the widespread adoption of low-osmolar contrast agents, the clinical impact of hydration has become less apparent. As Everson et al. noted, while the route, rate, and volume of fluids may have limited influence on outcome, maintaining euvolemia remains essential for at-risk patients [4]. The KDIGO guidelines also support continuing IV fluid administration over oral hydration for individuals at risk.

Our findings add to the evolving discourse around the safety of IV low-osmolar contrast use, particularly in hospitalized patients. We explored certain patients who appeared to be at greater risk. The study underscored the importance of tailored risk assessment in vulnerable populations.

Limitations

Several limitations of this study warrant consideration. First, this study focused exclusively on contrast administration during CT scans and did not include intra-arterial procedures such as cardiac catheterization, which typically need a significantly higher volume of contrast media. Additionally, as noted in our methods, the exact dosage of contrast media administered to each participant was not available in our data set, which might limit the ability to assess the dose-dependent relationship with the development of CA-AKI. Second, as the study focused exclusively on hospitalized patients, the population was inherently more acutely ill, potentially overestimating the real risk of CA-AKI compared to outpatients. Moreover, selection bias or confounding indication was a concern as the non-contrast CT group was older and had a higher burden of hypertension and renal dysfunction, suggesting that clinicians might have selectively avoided contrast in higher-risk patients. Although we utilized multivariable adjustment to control these, the potential for residual confounding could not be entirely excluded. Third, because contrast administration was determined by clinical indications rather than random assignment, patients undergoing contrast-enhanced CT may have differed systematically from those receiving non-enhanced imaging. This introduced potential confounding by indication. Fourth, the retrospective design not only introduced selection bias but also had additional limitations, including incomplete capture of important variables such as volume status, contrast volume administered, and use of nephrotoxic medications. Chemotherapy exposure among malignancy patients, a potential confounder, was not recorded. Fifth, almost all participants included in this cohort were Afro-Caribbean patients, and all were treated at a single tertiary hospital in Jamaica with access to advanced radiology, dialysis, and intensive care services. These may have mitigated CA-AKI severity and limited the generalizability of findings to the non-Black population and smaller or resource-limited areas in the region. Sixth, this study was limited by a lack of post-discharge follow-up data. Consequently, we were unable to assess long-term renal outcomes such as 30-day or 90-day mortality or the development of CKD after AKI.

## Conclusions

Use of a low-osmolar contrast agent, iopromide, was not independently associated with AKI following contrast-enhanced CT imaging. Risk stratification would be essential, and preventive measures remain necessary for high-risk patients. Further long-term longitudinal studies are necessary to better understand the true burden of newer radiocontrast agents and the development of CA-AKI.
